# Photocatalytic Degradation of Selected Pharmaceuticals Using g-C_3_N_4_ and TiO_2_ Nanomaterials

**DOI:** 10.3390/nano9091194

**Published:** 2019-08-23

**Authors:** Aneta Smýkalová, Barbora Sokolová, Kryštof Foniok, Vlastimil Matějka, Petr Praus

**Affiliations:** 1 Department of Chemistry, VŠB Technical University of Ostrava, 17. listopadu 2172/15, 700 33 Ostrava, Czech Republic; 2 Institute of Environmental technologies, VŠB Technical University of Ostrava, 17. listopadu 2172/15, 700 33 Ostrava, Czech Republic

**Keywords:** g-C_3_N_4_, TiO_2_, photocatalytic degradation, pharmaceuticals, paracetamol, ibuprofen, diclofenac

## Abstract

Exfoliated graphitic carbon nitride (g-C_3_N_4_) and two commercially available nanomaterials from titanium dioxide (P25 and CG300) were tested for the photocatalytic degradation of paracetamol (PAR), ibuprofen (IBU), and diclofenac (DIC). Prior to photocatalytic experiments, the nanomaterials were characterized by common methods, such as X-ray diffraction (XRD), UV–VIS diffuse reflectance spectroscopy (DRS), Fourier transformed infrared spectroscopy in attenuated total reflection mode (FTIR–ATR), transmission electron microscopy (TEM), physisorption of nitrogen, and dynamic vapor adsorption (DVS) of water. The sizes and specific surface area (SSA) of the TiO_2_ nanoparticles were 6 nm and 300 m^2^·g^−1^ for CG300 and 21 nm and 50 m^2^·g^−1^ for P25. The SSA of g-C_3_N_4_ was 140 m^2^·g^−1^. All photocatalytic experiments were performed under UV (368 nm), as well as VIS (446 nm) irradiation. TiO_2_ P25 was the most active photocatalyst under UV irradiation and g-C_3_N_4_ was the most active one under VIS irradiation. Photodegradation yields were evaluated by means of high performance liquid chromatography (HPLC) and reaction intermediates were identified using gas chromatography with mass detection (GC–MS). Paracetamol and ibuprofen were totally removed but the intermediates of diclofenac were observed even after 6 h of irradiation. Some intermediates, such as carbazole-1-acetic acid, 2,6-dichloraniline, and hydroxylated derivates of diclofenac were identified. This study showed that g-C_3_N_4_ is a promising photocatalyst for the degradation of pharmaceuticals in an aqueous environment, under visible light.

## 1. Introduction

The utilization of pharmaceutical products has been continually increasing all over the world. The presence of pharmaceuticals and their metabolites in water is beginning to be a serious problem for humans and animals [1,2,3,4,5,6,7]. These compounds get to waterbodies from various sources, such as disposals from hospitals and households, excretions by humans and animals, and it is important to develop new and effective technologies for their removal from wastewaters and the whole aquatic ecosystem. Numerous papers have been published on this topic in the last decade [8,9,10,11,12,13,14].

The effort to utilize photodegradation for the removal of pharmaceuticals is reflected in a number of scientific papers that often deal with TiO_2_ photocatalysts [15,16,17,18,19]. Among the groups of pharmaceuticals, non-steroidal, anti-inflammatory drugs are widely used and, thus, are now widely present in waterbodies. Their typical representatives are diclofenac, paracetamol, and ibuprofen; these compositions were photocatalytically degraded in TiO_2_ suspensions in some studies [20,21,22,23,24,25].

Recently, metal-free graphitic carbon nitride has attracted attention as a photocatalyst due to its narrow band gap of 2.7 eV (in comparison to 3.2 eV of TiO_2_). The properties and applications of g-C_3_N_4_ have been described in many comprehensive review papers e.g., [26,27,28,29,30,31,32]. One of the strategies for the enhancement of photocatalytic efficiency of g-C_3_N_4_ is exfoliation of its bulk structure. The exfoliation results in a higher specific surface area and, therefore, the photocatalytic activity of these g-C_3_N_4_ structures is improved significantly [33].

The utilization of g-C_3_N_4_ for the photocatalytic degradation of the above-mentioned anti-inflammatory pharmaceutical (DIC, PAR, IBU) has still not been widely investigated. There are only a few papers that refer to the degradation of diclofenac [34] and ibuprofen [35] by pure g-C_3_N_4_. The aim of this work was to study the photocatalytic degradation of DIC, PAR, and IBU by using exfoliated g-C_3_N_4_ in comparison with TiO_2_. For this purpose, the commercial photocatalysts TiO_2_ P25 and CG300 were employed.

## 2. Materials and Methods 

### 2.1. Materials and Reagents

Water was prepared by reverse osmosis (Aqua Osmotic; Czech Republic) and used for the preparation of all solutions. TiO_2_ P25 from Evonic Industries (Essen, Germany) and TiO_2_ CG300 from Precheza (Přerov, Czech Republic) were used without further treatment. TiO_2_ CG300 was specially prepared by a sulphate route with a high specific surface-area of 250–350 m^2^·g^−1^ (manufacturer’s data). Melamine for the preparation of g-C_3_N_4_, paracetamol (acetaminophen), diclofenac sodium salt, ibuprofen, and chloroacetic acid were purchased from Sigma-Aldrich (Darmstadt, Germany). Ortho-phosphoric acid (85%), ammonium hydroxide solution (25%), hydrochloric acid (35%), and ethanol were purchased from Lachner (Neratovice, Czech Republic); acetonitrile Chromapur GG and methanol Chromapur GG were purchased from BC-CHEMSERVIS (Rožnov pod Radhoštěm, Czech Republic). HPLC grade water was obtained using a water purification system MicroPure UV (ThermoScientific, Waltham, MA, USA). Diethyl ether was purchased from VWR Chemicals (Radnor, PA, USA), N,O-bis(trimethyl)trifluoroacetamide (BSTFA) with trimethylchlorosilane (TMCS) were purchased from Sigma-Aldrich (Darmstadt, Germany).

Stock standard solutions were prepared by dissolving accurately weighed quantity of analytes in 1 mL of methanol (1.0 mg·cm^−3^). Working standard solutions were prepared by diluting the stock standard solutions with water. The stock and working standard solutions were stored at 4 °C and protected from daylight.

### 2.2. Preparation of Exfoliated g-C_3_N_4_

Bulk g-C_3_N_4_ was prepared by heating melamine (10 g) at 550 °C for 4 h with a heating rate of 3 °C·min^−1^ in a ceramic crucible (30 mL, diameter 5 cm) covered with a lid in a muffle furnace. The crucible was cooled down out of the furnace to room temperature and then ground in an agate mortar to fine powder. Exfoliated graphitic carbon nitride (0.5 g) was prepared by heating of bulk g-C_3_N_4_ in a ceramic crucible (50 mL, diameter 8 cm) in a thin layer on a ceramic plate. g-C_3_N_4_ was heated for 3 h in air at 500 °C, with a heating rate of 10 °C·min^−1^ and then cooled down outside the furnace.

### 2.3. Materials Characterization

UV–VIS diffusion reflectance spectra in the range of 220–1400 nm were recorded by a spectrophotometer Shimadzu UV-2600 (Shimadzu Corp., Japan) with an integrated sphere attachment ISR-2600Plus at room temperature. Measured reflectance was transformed into the Kubelka–Munk function, as follows.

(1) $$F\left(R_{\infty }\right)=\frac{{\left(1-R_{\infty \;}\right)}^{2}}{2R_{\infty }},$$

where *R_∞_* is the diffuse reflectance from a semi-infinite layer. The DRS spectra were transformed to the dependencies of *(F(R∞)·hν*)^2^ on *hν* in order for us to obtain band-gap energies of the tested nanomaterials.

The specific surface area of each nanomaterial was measured by a device SORPTOMATIC 1990 series (ThermoScientific, Waltham, MA, USA). SSA was determined by the analysis of N_2_ adsorption isotherm at −196 °C by means of the Brunauer–Emmett–Teller (BET) method.

X-ray diffraction was performed using a Rigaku Ultima IV (Tokyo, Japan) diffractometer equipped with a Cu tube. The powder diffraction patterns of the studied nanomaterials were collected in a reflection mode in the range of 10–80 °2θ. The phase composition of the samples was evaluated using the Rigaku PDXL software with the PDF 2 database.

Fourier transform infrared spectroscopy was measured using the Nicolet iS50 device (ThermoScientific, Waltham, MA, USA). The spectra were collected in the ATR mode using a diamond ATR crystal. The spectra were collected in the wavenumber range of 400–4000 cm^−1^, 32 scans were averaged. The ATR correction followed by baseline subtracting was applied on each spectrum using the OMNIC software (Waltham, MA USA) and the final spectra were drawn in the Origin 8 Pro software (Northampton, MA, USA).

Transmission electron microscopy was performed with a JEOL 2100 microscope with (Jeol Ltd., Tokyo, Japan) a LaB6 electron gun. The accelerating voltage of 200 kV was applied. Micrographs were taken by a camera Tengra (EMSIS GmbH, Münster, Germany). For the TEM analysis, the samples were prepared by dispersion in ethanol and then were sonicated for 5 min. One drop of this solution was placed on a copper grid with a holey carbon film and was dried at room temperature.

Moisture adsorption on the nanomaterials was studied by using a dynamic vapor sorption system. The adsorption isotherms were recorded by weighing of samples under static humidity conditions, with the relative humidity being progressively increased (decreased) by 10%; all measurements were performed at 25 °C. A DVS device, model DVS-Advantage 1, purchased from Surface Measurement Systems Ltd. (London, UK) was employed for these experiments.

### 2.4. Photocatalytic Experiments and Analytical Methods

Photocatalysis was carried out in beakers placed on magnetic stirrers. The solutions were irradiated for a maximum of 6 h with 1 h in the dark to reach adsorption–desorption equilibria. A UVA lamp (368 nm) with an intensity of 0.96 mW cm^−2^ and a visible light emitting lamp (446 nm) with an intensity of 8.5 mW·cm^−2^ were used for the photodegradation experiments. The light intensity was measured using an optical power and energy meter PM200 (ThorLabs, Newton, NJ, USA) with a photodiode power sensor S120CV (ThorLabs). In a typical experiment, 0.9 g of a photocatalyst was suspended in PAR (25 g·dm^−3^), IBU (15 g·dm^−3^), or DIC (25 g·dm^−3^) solutions. Five beakers containing reaction suspensions were used for the chosen time intervals—before dark, after dark, 2 h, 4 h, and 6 h of exposure. Each reaction suspension was filtered using a syringe filter.

The filtered reaction solutions were analyzed by HPLC using a liquid chromatograph equipped with Nexera XR pumps (ThermoScientific, Waltham, MA, USA) and an SPD-M20A diode array detector (Shimadzu, Kyoto, Japan). Chromatographic separations were carried out on a Kinetex XB-C18 analytical column (150 × 4.6 mm, i.d. 2.6 μm) equipped with a guard column, using an isocratic mode. The mobile phase for the separation of ibuprofen and diclofenac sodium salts was a mixture of chloroacetic acid with ammonium hydroxide (pH 3) and acetonitrile 30/70 (*v*/*v*). The mobile phase for paracetamol was a mixture of 1.8 mmol·dm^−3^ ortho-phosphoric acid and acetonitrile 60/40 (*v*/*v*). The flow rate was 1.0 cm^3^·min^−1^. The chromatographic system operated at 25 °C. For quantitative analyses, selective detection was performed at 223 nm, 276 nm, and 246 nm for ibuprofen, diclofenac, and paracetamol, respectively.

GC–MS analyses were carried out on an 8890 GC system equipped with a single quad detector 5977B (Agilent Technologies, Santa Clara, CA, USA). An HP-5MS UI fused silica column (Agilent Tech., 30 m × 0.25 mm ID, 0.25 μm film thickness) was used. The flow rate of the He carrier gas was set at 1.1 cm^3^ min^−1^. The temperature program was as follows−70 °C for 1.5 min, 20 °C·min^−1^ up to 160 °C and hold time 0 min, 15 °C·min^−1^ up to 280 °C and hold time 10 min. Injector and transferline temperatures were 270 °C and 280 °C, respectively. The MS detector was operated in an electron ionization mode, scanning in the range of 50–600 amu.

Aliquots (10 mL) of the filtered reaction solutions were extracted to 15 mL diethyl ether for 5 min. Supernatants were dried under nitrogen and derivatized by a mixture of 25 µL of derivatization agents (BSTFA + TMCS) and 25 µL of ethyl acetate (70 °C, 30 min). After cooling, 1 µL of the sample was analyzed by GC–MS.

The photocatalytic degradations caused by the TiO_2_ nanomaterials were evaluated through absorbances of the pharmaceuticals measured by a Shimadzu UV-2600 spectrophotometer, using quartz cuvettes. The photodegradation using g-C_3_N_4_ was evaluated by HPLC (g-C_3_N_4_ particles in filtrates absorbed in the UV spectrum).

## 3. Results and Discussion

The tested nanomaterials were characterized by the common methods described in Section 2.3, such as UV–VIS DRS, XRD, FTIR–ATR spectrometry, and TEM. The specific surface area was measured by the physisorption of nitrogen and was evaluated by the BET method. The sorption of moisture was measured by DVS. After the characterization, the nanomaterials were employed for the photocatalytic degradation of the selected pharmaceuticals, using the procedure described in Section 2.4. It was possible to note that TiO_2_ CG300 was produced as a catalyst of the Claus reaction but its utilization as a photocatalyst could be expected.

### 3.1. Diffusion Reflectance Spectrometry

**Figure 1:**
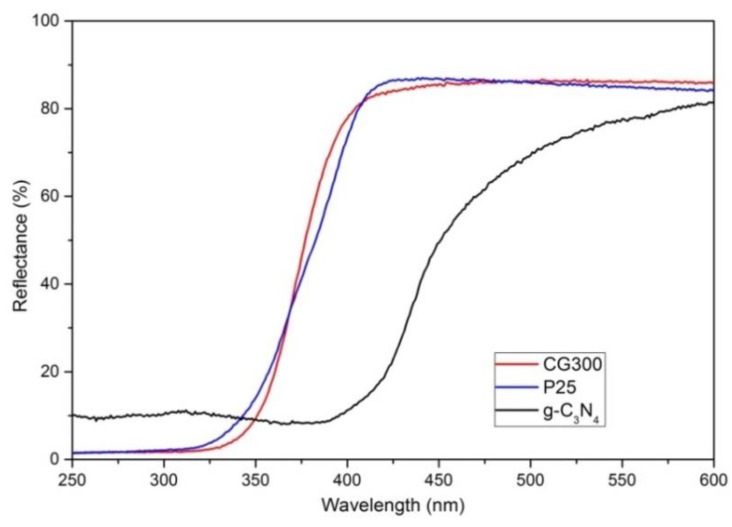
Diffusion reflectance spectra of the tested nanomaterials.

First, the ability of the nanomaterials to absorb UV and VIS light was examined by DRS. The DRS spectra were recorded (Figure 1) and further used for the determination of optical band gap energy (hereinafter, only band gap energy).

Unlike the reflectance of TiO_2_ samples, the reflectance of g-C_3_N_4_ was redshifted to the visible spectrum, as expected. The Kubelka–Munk functions *F(R_∞_)* were calculated from the reflectance values according to Equation (1) and the band gap energies were determined using the common method of Tauc´s plots, as follows:

(2) $$F\left(R\right)h\nu =C{\left(h\nu -E_{g}\right)}^{p},$$

where *hν* is the energy of incident photons, *E_g_* is the band gap energy, *C* is a constant, and *p* is the power depending on the type of electron transition. The power *p* = 2 and *p* = 0.5 are for direct and indirect semiconductors, respectively. Usually, the determination of *E_g_* involves plotting *(εhν)^1/p^* against *hν*. The determined band gap energies decreased in sequence 3.25 eV of CG300, 3.02 eV of P25, and 2.70 eV of g-C_3_N_4_. The different band gap energies of TiO_2_ were caused by their different crystallographic compositions, that is, the content of anatase and rutile. The band gap energies of pure anatase and rutile are reported in the literature as 3.2 eV and 3.0 eV, respectively [36]. The *E_g_* value of CG300 (3.25 eV) implies the presence of only anatase in CG300. The *E_g_* of 3.05 eV indicates the presence of rutile in P25. It was verified by the XRD analysis, as shown below.

### 3.2. X-ray Diffraction

**Figure 2:**
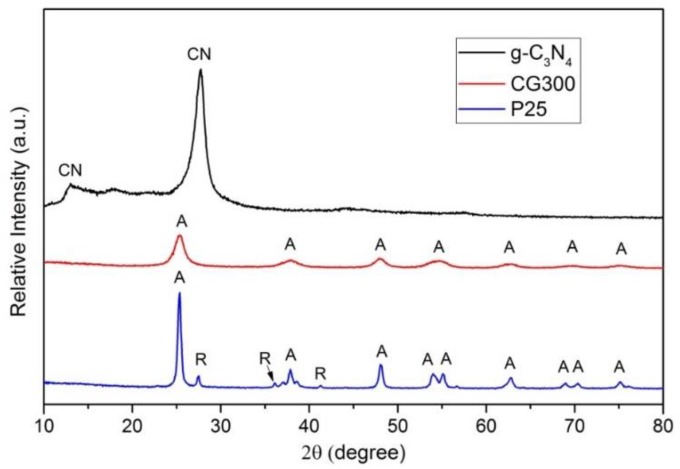
X-ray diffraction (XRD) patterns of the tested nanomaterials. A—anatase, R—rutile, CN—g-C_3_N_4._

The XRD patterns of the tested nanomaterials are displayed in Figure 2. A typical diffraction pattern of P25 shows the presence of both the anatase and rutile phases while the diffraction pattern of CG300 confirms the presence of only anatase. It was evident that in the case of CG300, the anatase diffraction peaks were broader than that in P25, which indicated a lower crystallite size of anatase in CG300. Its crystallite size was evaluated using the Halder–Wagner approach [37] implemented in the Rigaku PDXL software as *L* = 17 nm and *L* = 6 nm for P25 and CG300, respectively. The diffraction pattern registered for g-C_3_N_4_ showed the dominant diffraction peak centered at 27.75 °2θ, which was ascribed to the interlayer stacking of the aromatic (002) planes, and the less pronounced diffraction peak centered at 12.99 °2θ was ascribed to the in-plane structural packing of the triazine (100) plane [38].

### 3.3. FTIR-ATR Spectrometry

**Figure 3:**
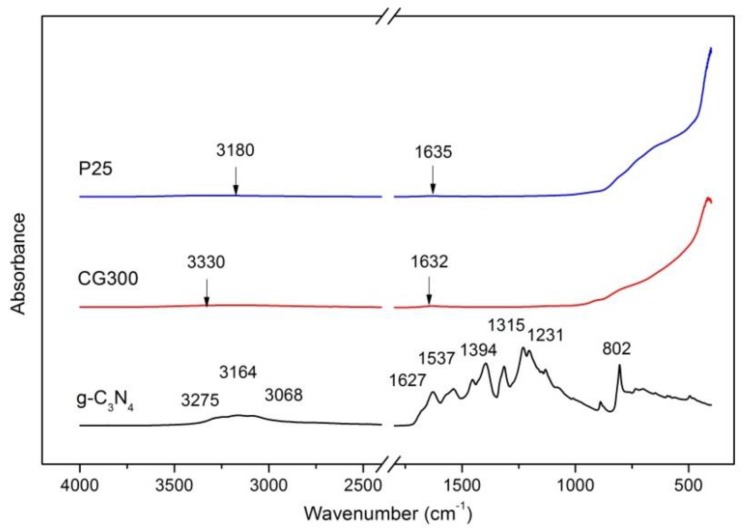
FTIR–ATR spectrum of the tested nanomaterials.

The FTIR-ATR spectra of the studied nanomaterials are shown in Figure 3. The presence of Ti-O bonds was evidenced in the area below 1000 cm^−1^ where absorption was ascribed to Ti-O stretching modes of anatase and rutile [39,40]. The bands at 3000–3800 cm^−1^ and 1630 cm^−1^ were ascribed to O-H stretching and bending vibrations, respectively, of the adsorbed water [41,42]. Detail views on the O-H vibrations are shown in Figures S1 and S2. 

The FTIR–ATR spectrum of g-C_3_N_4_ shows the presence of peaks in the region of 1200–1700 cm^−1^, which correspond to the stretching modes of aromatic CN heterocycles. The sharp peak at 802 cm^−1^ belonged to the characteristic breathing mode of s-triazine units and peaks in the region above 3000 cm^−1^ evidencing the stretching vibrations of N–H bonds [43,44].

### 3.4. TEM Analysis and Specific Surface Area

**Figure 4:**
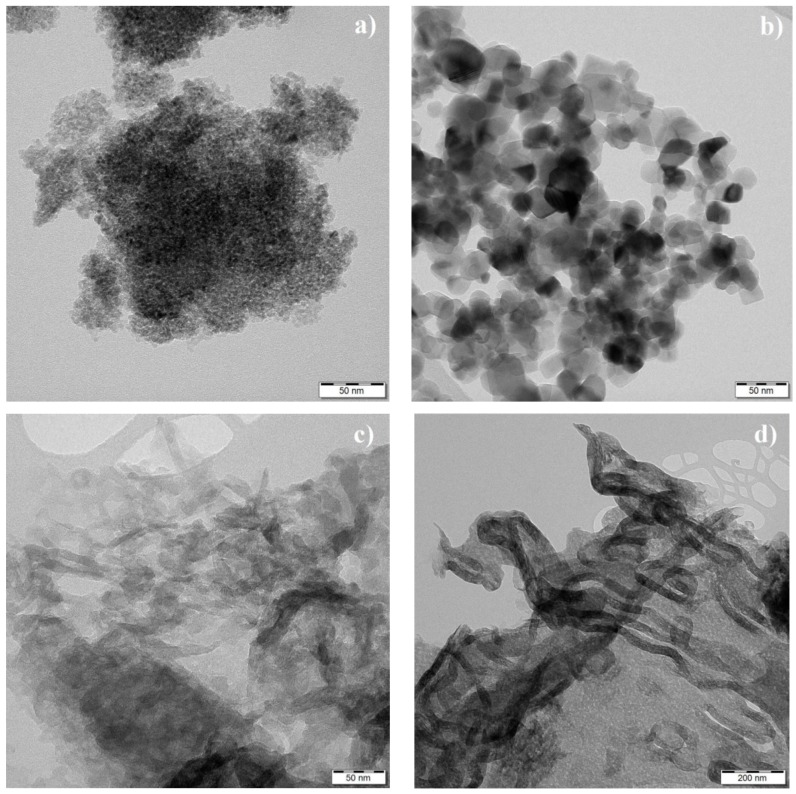
TEM micrographs of TiO_2_ and exfoliated g-C_3_N_4_. (**a**) TiO_2_ CG300, (**b**) TiO_2_ P25, and (**c**,**d**) g-C_3_N_4_.

The morphology and sizes of nanoparticles were investigated by means of transmission electron microscopy. The TEM micrographs are displayed in Figure 4. It is well visible that the CG300 shown in Figure 4a was formed from smaller nanoparticles than P25 in Figure 4b. Unlike the particle morphology of TiO_2_, the particle morphology of g-C_3_N_4_ was not so unambiguous, see Figure 4c,d. The complex structures were composed of flake-like sheets and snake-like shells. A shell-like structure, like that after thermal exfoliation, was observed for the g-C_3_N_4_ treated at high temperatures. Through thermal exfoliation, the flat nanosheets partially wrapped themselves into the shell-like structures. This was also observed in the decrease of the average g-C_3_N_4_ particle sizes, which were already measured by the dynamic light scattering (DLS) method [33].

The sizes of the TiO_2_ nanoparticles were estimated from the TEM micrographs. For both sorts of TiO_2_ nanoparticles, 91 subjects were evaluated by means of freely available ImageJ software. The sizes were found to be normally distributed and the average ones were computed at 4.0 nm for CG300 and 20.5 nm for P25. Summary statistics of the evaluated sizes are given in Table 1 and their histograms are shown in Figure S3.

The exfoliation of g-C_3_N_4_ was documented by the increase of its specific surface area. Through exfoliation, the SSA increased from 10 m^2^·g^−1^ (only bulk g-C_3_N_4_) to 140 m^2^·g^−1^. In the case of the TiO_2_ nanomaterials, the SSAs of 300 m^2^·g^−1^ and 50 m^2^·g^−1^ were determined for CG300 and P25, respectively. The basic characteristics of CG300 are high SSA and a low content of the remaining salts [45]. 

### 3.5. Moisture Adsorption

**Figure 5:**
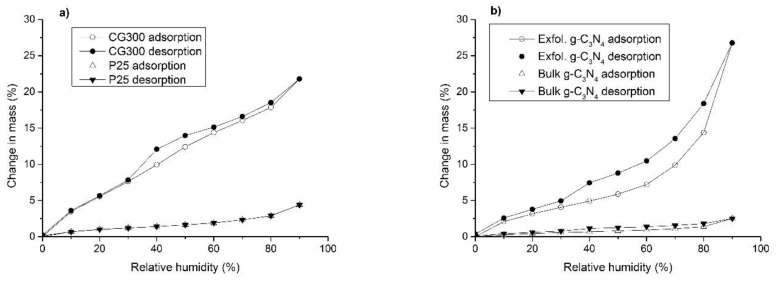
Moisture adsorption and desorption isotherm plots of TiO_2_ (**a**) and g-C_3_N_4_ (**b**).

Surface properties of the tested nanomaterials were also examined by the adsorption of moisture. Figure 5 shows the adsorption and desorption plots of water vapor for both TiO_2_ samples, exfoliated g-C_3_N_4_, and bulk g-C_3_N_4_, which were used for comparison.

The isotherms concerning the TiO_2_ nanomaterials demonstrated that CG300 absorbed more water than P25. Unlike the P25 plots, the CG300 plots exhibited a small hysteresis loop as well. These findings could be explained by a different phase composition of the TiO_2_ nanomaterials. The P25 was thought to have contained more than 70% of anatase, a minor amount of rutile, and a small amount of amorphous phase [46]. The manufacturer of CG300 it was composed of anatase. Our XRD analysis proved both pieces of information to be correct. First-principles molecular dynamics calculations predicted the dissociative adsorption of water molecules on the rutile (110) surface. Non-dissociated water molecules were adsorbed to anatase through H bonds to the Ti-OH species of the (101) surface [47] or were coordinated to the Ti^4+^ cations [48]. The DVS experiments confirmed these theoretical hypotheses, as displayed in Figure 5.

The isotherms of exfoliated g-C_3_N_4_ indicated a larger amount of adsorbed water than what was adsorbed on bulk g-C_3_N_4_. The hysteresis loop of exfoliated g-C_3_N_4_ was also larger, likely due to the adsorption of water through H bonds to the terminating =NH and –NH_2_ groups, which were better assessable in the exfoliated structures.

### 3.6. Photocatalytic Degradation of Selected Pharmaceuticals

**Figure 6:**
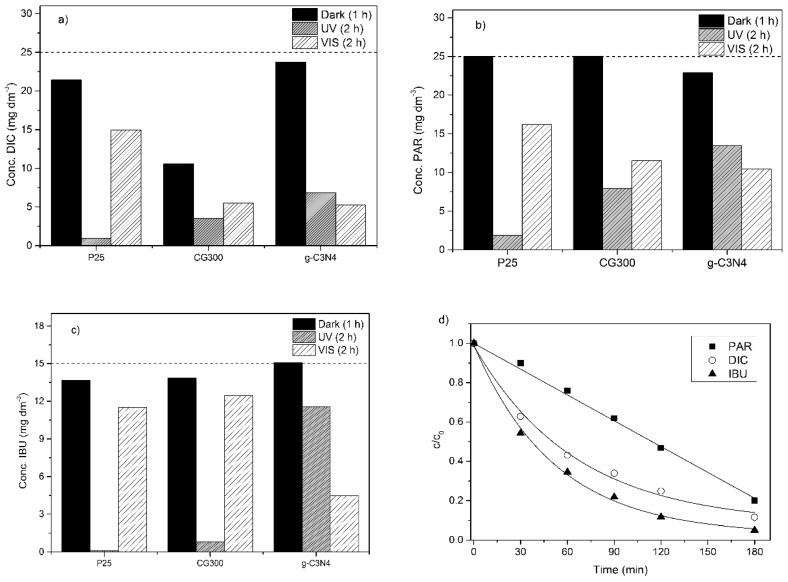
Graphs of photocatalytic degradation of pharmaceuticals after 2 h of irradiation. (**a**) Diclofenac (DIC) under UV light; (**b**) paracetamol (PAR) under VIS light; (**c**) ibuprofen (IBU) under UV light; (**d**) reaction curves of PAR, DIC, and IBU in the presence of g-C_3_N_4_ under VIS irradiation.

Both sorts of TiO_2_ nanomaterials as well as exfoliated g-C_3_N_4_ were tested for the photocatalytic degradation of paracetamol, ibuprofen, and diclofenac. First, their aqueous suspensions were irradiated by UV and VIS light for 2 h, see Figure 6a–c and Table 2. The photocatalytic experiments started after the adsorption–desorption equilibrium between the pharmaceuticals when the nanomaterials were established (after dark). No photolysis of the pharmaceuticals was observed. 

Under the UV irradiation, the photodegradation process of ibuprofen produced a noticeable odor, which has not been described in the literature yet. During the diclofenac photodegradation, a slightly pinkish solution was observed after 2–3 h, which vanished with a longer irradiation time, due to the degradation of intermediate products. In the case of paracetamol, no special effect was observed. The degradation efficiency of photocatalysts for all pharmaceuticals increased in sequence g-C_3_N_4_ < CG300 < P25. This was not surprising when taking into account the wavelength of 368 nm and the band gap energies of the TiO_2_ nanomaterials. The lowest photodegradation activity of g-C_3_N_4_ was likely due to its fast photoinduced electron-hole recombination. The interesting observation was that about six-times higher SSA rate of the CG300 as compared to the P25 did not affect its photodegradation efficiency. This could be explained by a synergistic effect of anatase and rutile phase mixture of P25, which led to the improved separation of photoinduced electrons and holes [49,50].

During the VIS irradiation the degradation efficiency increased in sequence P25 < CG300 < g-C_3_N_4_. The degradation efficiency using g-C_3_N_4_ of about 77% (VIS) and 7% (UV) were in agreement with the band gap energy of 2.70 eV (459 nm). Therefore, g-C_3_N_4_ should be active under UV (368 nm) as well as under VIS (446 nm). The photodegradation in the presence of TiO_2_ could not be attributed to sensitization by the adsorbed pharmaceuticals, which was often observed in the case of various dyes [51,52], because none of these compounds absorbed visible light at 446 nm, see Figure S4. The probable reason was the VIS light absorption of both TiO_2_ nanomaterials, as a result of their structural defects, such as oxygen vacancies [53,54,55]. The oxygen vacancies are types of intrinsic defects forming intermediate energy levels within the TiO_2_ band gap [56], which act as recombination centers for photoinduced electrons and holes [57]. 

**Figure 7:**
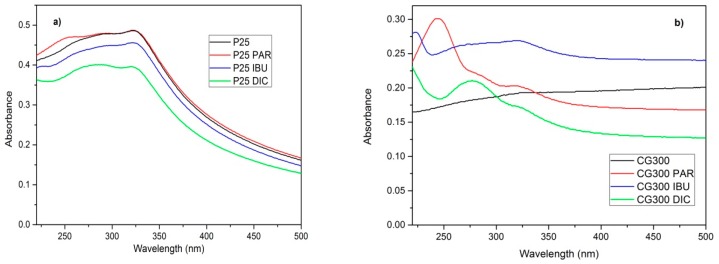
Absorption spectra of suspensions of P25 (**a**) and CG300 (**b**) and pharmaceuticals before photocatalytic reactions.

Figure 7 displays the absorption spectra of TiO_2_ suspensions of both pure TiO_2_ nanomaterials and their mixtures, with the pharmaceuticals. The suspension of P25 was diluted 60 times and that of CG300 was diluted 15 times in order for us to correctly obtain the measurable absorbances. The spectra were recorded after 1 h in the dark, before the start of the photocatalytic reactions. In both cases, the TiO_2_ absorbed visible light likely due to electron transfers between their valence bands and the intermediate energy levels of oxygen vacancies mentioned above. Absorption bands of the pure pharmaceuticals (see Figure S4) superposed with a large band of TiO_2_ in the CG300 spectrum were quite obvious. In the P25 suspension spectra, only hints of the pharmaceuticals were visible due to strong absorption of this TiO_2_. Therefore, the photodegradation of pharmaceuticals proceeded only by photocatalysis and not by sensitization, which is often referred to in the literature. As a result, photocatalysis was possible even if *hν < E_g._*

The original concentrations of the pharmaceuticals in Figure 6 are highlighted by dashed lines to demonstrate their adsorption to the nanomaterials. As can be seen, paracetamol was not adsorbed by the TiO_2_ nanomaterials regardless of their types and SSA but was adsorbed by g-C_3_N_4_ likely due to van der Waals’s interactions between the aromatic rings of paracetamol and heptazine units of g-C_3_N_4_. On the contrary, ibuprofen and diclofenac were adsorbed by TiO_2_ but ibuprofen was not adsorbed by g-C_3_N_4_. The interactions of IBU hydroxyl and carbonyl groups with the g-C_3_N_4_ planes were supposed to be unimportant. However, in the case of TiO_2_, these interactions were important due to the polar character of the Ti–O bonds. Diclofenac was adsorbed to g-C_3_N_4_ more than ibuprofen but less than paracetamol. The complex van der Waals’s polar and nonpolar interactions between DIC and g-C_3_N_4_ were also assumed. 

The adsorption of diclofenac to CG300 was not clear. As demonstrated by the DVS isotherms, the CG300 nanoparticles adsorbed many water molecules, which would have likely pushed the DIC hydrophobic molecules (logK_OW_ = 4.51 [3] or 1.90 [58]) back into the aqueous suspension [59]. However, diclofenac is a sodium salt and its negative charge is supposed to interact with the Ti^4+^ cations of the CG300 surface, causing a huge adsorption.

#### Evaluation of Photodegradation Kinetics

Photocatalytic degradations are supposed to be based on the reactions of hydroxyl radicals formed by complex reactions of photoinduced electrons and holes (h) with oxygen, water, and hydroxide ions [60,61,62,63], as follows:

h^+^ + H_2_O → OH^•^ + H^+^, (3)

h^+^ + OH^−^ → OH^•^, (4)

e^−^ + O_2_ → O_2_^•−^, (5)

O_2_^•−^ + H^+^ ⇆ HO_2_^•^ (pK_a_ = 4.8), (6)

2 HO_2_^•^ → H_2_O_2_ + O_2_, (7)

H_2_O_2_ + e^−^ → OH^•^ + OH^−^, (8)

Heterogeneous reactions of the pharmaceuticals and hydroxyl radicals on the surface of g-C_3_N_4_ and TiO_2_ can be described by the Langmuir–Hinshelwood model [62], as follows:

(9) $$r=-\frac{dc_{P}}{dt}=k\frac{K_{P}c_{P}}{1+K_{P}c_{P}+\sum^{\;}K_{i}c_{i}}\frac{K_{OH}\;c_{OH}}{1+K_{OH}c_{OH}}$$

where *k* is the kinetic parameter; *K_P_*, *K_OH_*, *K_i_* and *c_P_*, *c_O_*_H_, *c_i_* are the adsorption constants and the concentrations of the remaining pharmaceuticals, hydroxyl radicals, and intermediates, respectively. If *c_OH_* >> *c_P_* and *ΣK_i_c_i_* is neglected then Equation (9) can be simplified to its most commonly used form:

(10) $$r=-\frac{dc_{P}}{dt}=k_{app}\frac{K_{P}c_{P}}{1+K_{P}c_{P}},$$

where *k_app_* is the apparent kinetic parameter depending on the irradiation intensity, mass, and nature of a solid phase (photocatalyst) and the concentration of the OH^•^ radicals. This model was theoretically approved to be appropriate to the first-order kinetics [64]. On the contrary, if *c_OH_* << *c_P_* and *ΣK_i_c_i_* was neglected then Equation (9) could be simplified to:

(11) $$r=-\frac{dc_{P}}{dt}=k_{app}\frac{K_{OH}c_{OH}}{1+K_{OH}c_{OH}}=k_{obs},$$

where *k_obs_* is the observed kinetic constant, supposing that the concentration of hydroxyl radicals is constant. Then, the reaction rate is also constant and the decomposition of pharmaceuticals is of the zero-order reaction. The constant concentration of hydroxyl radicals is possible when an irradiating flux is constant, a photocatalyst is stable, and its surface sites are not occupied by intermediates. 

In this study, g-C_3_N_4_ was found to be the most active photocatalyst under VIS irradiation, which is very promising for its future environmental applications. The reaction kinetic curves of the photocatalytic degradation of all pharmaceuticals are shown in Figure 6d. The degradation of ibuprofen and diclofenac followed the first-order kinetics, which is common for most of the degradation reactions. However, paracetamol obeyed the zero-order reaction kinetics. The kinetic constants were evaluated as *k_app_* = 5.5 ± 1.1 × 10^−3^ (min^−1^) for ibuprofen and a higher *k_app_* = 7.2 ± 0.6 × 10^−3^ (min^−1^) was observed for diclofenac. The kinetic constant *k_obs_* for paracetamol was 4.5 ± 0.3 × 10^−3^ (mol·dm^−3^·min^−1^).

### 3.7. Photodegradation and Analysis of Intermediates

**Figure 8:**
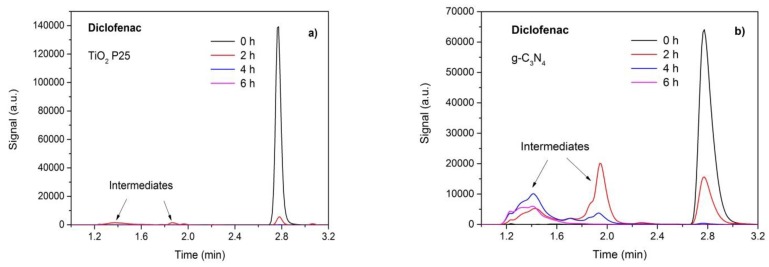
Photocatalytic degradation of diclofenac using TiO_2_ P25 under UV (**a**) and exfoliated g-C_3_N_4_ (**b**) under VIS irradiation.

The photodegradation of pharmaceuticals as well as the resulting products—intermediates—were analyzed by HPLC after 2 h, 4 h, and 6 h of the UV–VIS irradiation. Using TiO_2_ P25 as the most active photocatalyst under UV irradiation, no intermediates of paracetamol and ibuprofen were observed. Only in the case of diclofenac, small amounts of some intermediates were found at the retention time of about 1.1–2.2 min, as demonstrated in Figure 8a.

Figure 8b displays the chromatograms of diclofenac decomposed in the presence of g-C_3_N_4_ under VIS irradiation. Intermediates resulting after 2 and 4 h were observed in two broad peaks migrating in front of the peaks of diclofenac (at 2.8 min). The first unresolved peak with the retention time of 1.2–1.6 min contained the final degradation products, which was obvious from the chromatogram after 6 h of irradiation. The second peak between 1.8 min and 2.2 min contained intermediates, which were further transformed. It indicated that the photodegradation process consisted of several steps, during which diclofenac was not completely decomposed but some unknown intermediates remained in the reaction mixture. The photocatalytic degradation did not remove the intermediates even after 12 h (Figure S4) and, therefore, the new experimental conditions of the photocatalysis will be investigated in the future experiments.

The photodegradation intermediates of diclofenac were identified by means of GC–MS. Carbazole-1-acetic acid was found as a product of losing chlorine by the absorption of photons [65] or elimination of hydrochloric acid and photoreduction [66]. Other intermediates were formed by various reactions with hydroxyl radicals. 2,6-dichloraniline might arose from oxidation at the nitrogen atom of diclofenac [67]. Several hydroxylated derivates of diclofenac, such as 2-[(2,6-dichlorophenyl)amino]-5-hydroxyphenylacetic acid were identified as well.

## 4. Conclusions

Exfoliated g-C_3_N_4_ and commercially available nanoparticles of titanium dioxide P25 and CG300 were investigated for the photocatalytic degradation of paracetamol, ibuprofen, and diclofenac. Exfoliated g-C_3_N_4_ was prepared by thermal synthesis of bulk g-C_3_N_4_ from melamine at 550 °C and by consequent exfoliation at 500 °C. The TiO_2_ nanoparticles were of different phase composition and SSA. P25 consisted of rutile and anatase, and CG300 was formed only from anatase. The SSA values (the BET method) were 300 m^2^·g^−1^ and 50 m^2^·g^−1^ for CG300 and P25, respectively. The CG300 and P25 nanoparticle sizes were 4 nm and 21 nm, respectively. The SSA of g-C_3_N_4_ was 140 m^2^·g^−1^. The band gap energies of CG300, P25, and g-C_3_N_4_ were 3.25 eV, 3.02 and 2.70 eV, respectively.

The adsorption of moisture measured by the DVS method agreed with information in the literature, pointing out that non-dissociative adsorption of water molecules on anatase was preferred to dissociative adsorption on rutile. Water was adsorbed to g-C_3_N_4_ likely through the hydrogen bonds to the =NH and –NH_2_ groups, which was deduced from the DVS experiments with bulk and exfoliated g-C_3_N_4_.

The photocatalytic experiments showed that P25 was the most active photocatalyst under UV irradiation and g-C_3_N_4_ was the most active one under VIS irradiation. Paracetamol and ibuprofen were totally removed but the intermediates of diclofenac were observed, even after 12 h of irradiation. Some intermediates, such as carbazole-1-acetic acid, 2,6-dichloraniline, and hydroxylated derivates of diclofenac, were identified by GC–MS as displayed in Figure S5. 

Our next experiments will be focused on searching for appropriate photocatalytic conditions for the total and fast degradation of diclofenac and other pharmaceuticals, such as various antibiotics, hormones, and drugs.

## Figures and Tables

**Table 1:** Basic statistics of TiO_2_ nanoparticles.

Statistic	TiO_2_ CG300	TiO_2_ P25
Minimum (nm)	2.3	10.6
Maximum (nm)	6.0	32.6
Average (nm)	4.0	20.5
Standard deviation (nm)	0.83	5.1
Average confidence interval (nm)	3.8–4.2	19.5–21.6
Skewness	0.121	0.341
Kurtosis	2.53	2.65

**Table 2:** Photodegradation efficiency of pharmaceuticals after 2 h of irradiation.

Pharmaceutical	Photodegradation (%)	
	Paracetamol	
	VIS	UV
P25	35	93
CG300	54	69
g-C_3_N_4_	54	41
	Ibuprofen	
P25	17	99
CG300	13	94
g-C_3_N_4_	71	24
	Diclofenac	
P25	25	96
CG300	49	66
g-C_3_N_4_	77	73

## Supplementary Materials

The following are available online at https://www.mdpi.com/2079-4991/9/9/1194/s1. Figure S1. FTIR–ATR spectrum of TiO_2_ P25. Figure S2. FTIR–ATR spectrum of TiO_2_ CG300. Figure S3. Histograms for the sizes of the TiO_2_ nanoparticles. (a) CG300 and (b) P25. Figure S4. Absorption spectra of DIC, IBU and PAR in the concentration of 20 mg/L. Figure S5. HPLC chromatograms of diclofenac photodegradation with g-C_3_N_4_.

## References

[1] Barbosa M.O., Moreira N.F.F., Ribeiro A.R., Pereira M.F.R., Silva A.M.T. (2016). Occurrence and removal of organic micropollutants: An overview of the watch list of EU Decision 2015/495. Water Res..

[2] Fekadu S., Alemayehu E., Dewil R., Van der Bruggen B. (2019). Pharmaceuticals in freshwater aquatic environments: A comparison of the African and European challenge. Sci. Total Environ..

[3] Manvendra P., Kumar R., Kishor K., Mlsna T., Pittman C.U., Mohan D. (2019). Pharmaceuticals of Emerging Concern in Aquatic Systems: Chemistry, Occurrence, Effects, and Removal Methods. Chem. Rev..

[4] Ojemaye C.Y., Petrik L. (2018). Pharmaceuticals in the marine environment: A review. Environ. Rev..

[5] Kim M.-K., Zoh K.-D. (2016). Occurrence and removals of micropollutants in water environment. Environ. Eng. Res..

[6] Yang Y., Ok Y.S., Kim K.-H., Kwon E.E., Tsang Y.F. (2017). Occurrences and removal of pharmaceuticals and personal care products (PPCPs) in drinking water and water/sewage treatment plants: A review. Sci. Total Environ..

[7] Petrie B., Barden R., Kasprzyk-Hordern B. (2015). A review on emerging contaminants in wastewaters and the environment: Current knowledge, understudied areas and recommendations for future monitoring. Water Res..

[8] Quesada H.B., Baptista A.T.A., Cusioli L.F., Seibert D., de Oliveira Bezerra C., Bergamasco R. (2019). Surface water pollution by pharmaceuticals and an alternative of removal by low-cost adsorbents: A review. Chemosphere.

[9] Xu Y., Liu T., Zhang Y., Ge F., Steel R.M., Sun L. (2017). Advances in technologies for pharmaceuticals and personal care products removal. J. Mater. Chem. A.

[10] Tiwari B., Sellamuthu B., Ouarda Y., Drogui P., Tyagi R.D., Buelna G. (2017). Review on fate and mechanism of removal of pharmaceutical pollutants from wastewater using biological approach. Bioresour. Technol..

[11] Kanakaraju D., Glass B.D., Oelgemöller M. (2018). Advanced oxidation process-mediated removal of pharmaceuticals from water: A review. J. Environ. Manag..

[12] Elisa L., Edgar M., Kim M.B., Saul N., Elvira Z. (2018). A Review on Chemical Advanced Oxidation Processes for Pharmaceuticals with Paracetamol as a Model Compound. Reaction Conditions, Intermediates and Total Mechanism. Curr. Org. Chem..

[13] Rivera-Utrilla J., Sánchez-Polo M., Ferro-García M.Á., Prados-Joya G., Ocampo-Pérez R. (2013). Pharmaceuticals as emerging contaminants and their removal from water. A review. Chemosphere.

[14] Esplugas S., Bila D.M., Krause L.G.T., Dezotti M. (2007). Ozonation and advanced oxidation technologies to remove endocrine disrupting chemicals (EDCs) and pharmaceuticals and personal care products (PPCPs) in water effluents. J. Hazard. Mater..

[15] Kanakaraju D., Glass B., Oelgemöller M. (2014). Titanium dioxide photocatalysis for pharmaceutical wastewater treatment. Environ. Chem. Lett..

[16] Awfa D., Ateia M., Fujii M., Johnson M.S., Yoshimura C. (2018). Photodegradation of pharmaceuticals and personal care products in water treatment using carbonaceous-TiO_2_ composites: A critical review of recent literature. Water Res..

[17] Durán A., Monteagudo J.M., San Martín I. (2018). Operation costs of the solar photo-catalytic degradation of pharmaceuticals in water: A mini-review. Chemosphere.

[18] Sarkar S., Das R., Choi H., Bhattacharjee C. (2014). Involvement of process parameters and various modes of application of TiO_2_ nanoparticles in heterogeneous photocatalysis of pharmaceutical wastes – a short review. RSC Adv..

[19] Tong A., Braund R., Warren D., Peake B. (2012). TiO_2_-assisted photodegradation of pharmaceuticals—A review. Open Chem..

[20] Calza P., Sakkas V.A., Medana C., Baiocchi C., Dimou A., Pelizzetti E., Albanis T. (2006). Photocatalytic degradation study of diclofenac over aqueous TiO_2_ suspensions. Appl. Catal. B.

[21] Martínez C., Canle L.M., Fernández M.I., Santaballa J.A., Faria J. (2011). Aqueous degradation of diclofenac by heterogeneous photocatalysis using nanostructured materials. Appl. Catal. B.

[22] Rimoldi L., Meroni D., Falletta E., Ferretti A.M., Gervasini A., Cappelletti G., Ardizzone S. (2017). The role played by different TiO_2_ features on the photocatalytic degradation of paracetamol. Appl. Surf. Sci..

[23] Yang L., Yu L.E., Ray M.B. (2008). Degradation of paracetamol in aqueous solutions by TiO_2_ photocatalysis. Water Res..

[24] Candido J.P., Andrade S.J., Fonseca A.L., Silva F.S., Silva M.R.A., Kondo M.M. (2016). Ibuprofen removal by heterogeneous photocatalysis and ecotoxicological evaluation of the treated solutions. Environ. Sci. Pollut. Res. Int..

[25] Choina J., Kosslick H., Fischer C., Flechsig G.U., Frunza L., Schulz A. (2013). Photocatalytic decomposition of pharmaceutical ibuprofen pollutions in water over titania catalyst. Appl. Catal. B.

[26] Zhu B., Zhang L., Cheng B., Yu J. (2018). First-principle calculation study of tri-s-triazine-based g-C3N4: A review. Appl. Catal. B.

[27] Zhou Z., Zhang Y., Shen Y., Liu S., Zhang Y. (2018). Molecular engineering of polymeric carbon nitride: Advancing applications from photocatalysis to biosensing and more. Chem. Soc. Rev..

[28] Wang Y., Wang X., Antonietti M. (2012). Polymeric graphitic carbon nitride as a heterogeneous organocatalyst: From photochemistry to multipurpose catalysis to sustainable chemistry. Angew. Chem. Int. Edit..

[29] Ong W.-J., Tan L.-L., Ng Y.H., Yong S.-T., Chai S.-P. (2016). Graphitic Carbon Nitride (g-C3N4)-Based Photocatalysts for Artificial Photosynthesis and Environmental Remediation: Are We a Step Closer To Achieving Sustainability?. Chem. Rev..

[30] Cao S., Low J., Yu J., Jaroniec M. (2015). Polymeric photocatalysts based on graphitic carbon nitride. Adv. Mater..

[31] Moniz S.J.A., Shevlin S.A., Martin D.J., Guo Z.-X., Tang J. (2015). Visible-light driven heterojunction photocatalysts for water splitting – a critical review. Energy Environ. Sci..

[32] Zhao Z., Sun Y., Dong F. (2015). Graphitic carbon nitride based nanocomposites: A review. Nanoscale.

[33] Svoboda L., Praus P., Lima M.J., Sampaio M.J., Matýsek D., Ritz M., Dvorský R., Faria J.L., Silva C.G. (2018). Graphitic carbon nitride nanosheets as highly efficient photocatalysts for phenol degradation under high-power visible LED irradiation. Mater. Res. Bull..

[34] Moreira N.F.F., Sampaio M.J., Ribeiro A.R., Silva C.G., Faria J.L., Silva A.M.T. (2019). Metal-free g-C3N4 photocatalysis of organic micropollutants in urban wastewater under visible light. Appl. Catal. B.

[35] Hernández-Uresti D.B., Vázquez A., Sanchez-Martinez D., Obregón S. (2016). Performance of the polymeric g-C3N4 photocatalyst through the degradation of pharmaceutical pollutants under UV–vis irradiation. J. Photochem. Photobiol. A.

[36] Castrejón-Sánchez V.H., López R., Ramón-González M., Enríquez-Pérez Á., Camacho-López M., Villa-Sánchez G. (2018). Annealing Control on the Anatase/Rutile Ratio of Nanostructured Titanium Dioxide Obtained by Sol-Gel. Crystals.

[37] Halder N.C., Wagner C.N.J. (1966). Separation of particle size and lattice strain in integral breadth measurements. Acta Crystallogr..

[38] Fina F., Callear S.K., Carins G.M., Irvine J.T.S. (2015). Structural Investigation of Graphitic Carbon Nitride via XRD and Neutron Diffraction. Chem. Mater..

[39] Kakuma Y., Nosaka A.Y., Nosaka Y. (2015). Difference in TiO_2_ photocatalytic mechanism between rutile and anatase studied by the detection of active oxygen and surface species in water. Phys. Chem. Chem. Phys..

[40] Haque F.Z., Nandanwar R., Singh P. (2017). Evaluating photodegradation properties of anatase and rutile TiO_2_ nanoparticles for organic compounds. Optik.

[41] Wang Y., Ganeshraja A.S., Jin C., Zhu K., Wang J. (2018). One-pot synthesis visible-light-active TiO_2_ photocatalysts at low temperature by peroxotitanium complex. J. Alloys Compd..

[42] Luo S., Wang F., Shi Z., Xin F. (2009). Preparation of highly active photocatalyst anatase TiO_2_ by mixed template method. J. Sol-Gel Sci. Technol..

[43] Praus P., Svoboda L., Ritz M., Troppová I., Šihor M., Kočí K. (2017). Graphitic carbon nitride: Synthesis, characterization and photocatalytic decomposition of nitrous oxide. Mater. Chem. Phys..

[44] Papailias I., Giannakopoulou T., Todorova N., Demotikali D., Vaimakis T., Trapalis C. (2015). Effect of processing temperature on structure and photocatalytic properties of g-C3N4. Appl. Surf. Sci..

[45] Precheza PRETIOX Titanium Dioxide CG300 for catalytic preparation. https://www.precheza.cz/root/ke-stazeni/katalogy/letak-pretiox-titcg300-screen.pdf.

[46] Ohtani B., Prieto-Mahaney O.O., Li D., Abe R. (2010). What is Degussa (Evonik) P25? Crystalline composition analysis, reconstruction from isolated pure particles and photocatalytic activity test. J. Photochem. Photobiol. A.

[47] Selloni A., Vittadini A., Grätzel M. (1998). The adsorption of small molecules on the TiO_2_ anatase (101) surface by first-principles molecular dynamics. Surf. Sci..

[48] Bolis V., Busco C., Ciarletta M., Distasi C., Erriquez J., Fenoglio I., Livraghi S., Morel S. (2012). Hydrophilic/hydrophobic features of TiO_2_ nanoparticles as a function of crystal phase, surface area and coating, in relation to their potential toxicity in peripheral nervous system. J. Colloid Interface Sci..

[49] Ohno T., Tokieda K., Higashida S., Matsumura M. (2003). Synergism between rutile and anatase TiO_2_ particles in photocatalytic oxidation of naphthalene. Appl. Catal. A.

[50] Zhang Y., Gan H., Zhang G. (2011). A novel mixed-phase TiO_2_/kaolinite composites and their photocatalytic activity for degradation of organic contaminants. Chem. Eng. J..

[51] Rochkind M., Pasternak S., Paz Y. (2014). Using dyes for evaluating photocatalytic properties: A critical review. Molecules.

[52] Konstantinou I.K., Albanis T.A. (2004). TiO_2_-assisted photocatalytic degradation of azo dyes in aqueous solution: Kinetic and mechanistic investigations: A review. Appl. Catal. B.

[53] Kuznetsov V.N., Serpone N. (2006). Visible Light Absorption by Various Titanium Dioxide Specimens. J. Phys. Chem. B.

[54] Kuznetsov V.N., Serpone N. (2009). On the Origin of the Spectral Bands in the Visible Absorption Spectra of Visible-Light-Active TiO_2_ Specimens Analysis and Assignments. J. Phys. Chem. C.

[55] Janotti A., Varley J.B., Rinke P., Umezawa N., Kresse G., Van de Walle C.G. (2010). Hybrid functional studies of the oxygen vacancy in TiO_2_. Phys. Rev. B.

[56] Nakamura I., Negishi N., Kutsuna S., Ihara T., Sugihara S., Takeuchi K. (2000). Role of oxygen vacancy in the plasma-treated TiO_2_ photocatalyst with visible light activity for NO removal. J. Mol. Catal. A Chem..

[57] Kernazhitsky L., Shymanovska V., Gavrilko T., Naumov V., Fedorenko L., Kshnyakin V., Baran J. (2014). Room temperature photoluminescence of anatase and rutile TiO_2_ powders. J. Lumin..

[58] Scheytt T., Mersmann P., Lindstädt R., Heberer T. (2005). 1-Octanol/Water Partition Coefficients of 5 Pharmaceuticals from Human Medical Care: Carbamazepine, Clofibric Acid, Diclofenac, Ibuprofen, and Propyphenazone. Water Air Soil Pollut..

[59] Praus P., Veteška M., Pospíšil M. (2011). Adsorption of phenol and aniline on natural and organically modified montmorillonite: Experiment and molecular modelling. Mol. Simul..

[60] Bhatkhande D.S., Pangarkar V.G., Beenackers A.A.C.M. (2002). Photocatalytic degradation for environmental applications – a review. J. Chem. Technol. Biotechnol..

[61] Mills A., Le Hunte S. (1997). An overview of semiconductor photocatalysis. J. Photochem. Photobiol. A.

[62] Brosillon S., Lhomme L., Vallet C., Bouzaza A., Wolbert D. (2008). Gas phase photocatalysis and liquid phase photocatalysis: Interdependence and influence of substrate concentration and photon flow on degradation reaction kinetics. Appl. Catal. B.

[63] Wood P.M. (1988). The potential diagram for oxygen at pH 7. Biochem. J.

[64] Kumar K.V., Porkodi K., Rocha F. (2008). Langmuir–Hinshelwood kinetics – A theoretical study. Catal. Commun..

[65] Moore D.E., Roberts-Thomson S., Zhen D., Duke C.C. (1990). PHOTOCHEMICAL STUDIES ON THE ANTIINFLAMMATORY DRUG DICLOFENAC. Photochem. Photobiol..

[66] Poiger T., Buser H.-R., Müller M.D. (2001). Photodegradation of the pharmaceutical drug diclofenac in a lake: Pathway, field measurements, and mathematical modeling. Environ. Toxicol. Chem..

[67] Vogna D., Marotta R., Napolitano A., Andreozzi R., d’Ischia M. (2004). Advanced oxidation of the pharmaceutical drug diclofenac with UV/H_2_O_2_ and ozone. Water Res..

