# Supplementary material for: Photocatalytic Degradation of Selected Pharmaceuticals Using g-C3N4 and TiO2 Nanomaterials
Source: Nanomaterials (Basel). 2019 Aug 23;9(9):1194. doi: 10.3390/nano9091194 (PMC6780102; doi:10.3390/nano9091194)
Supplement: Supplementary file 1 [file nanomaterials-09-01194-s001.pdf]

nanomaterials

Article

Photocatalytic Degradation of Selected Pharmaceuticals by Using g-C_3N_4 and TiO_2 Nanomaterials

Aneta Smýkalová [1,2], Barbora Sokolová [2], Kryštof Foniok[1], Vlastimil Matějka[1,2] and Petr Praus[1,2,*]

[1] Department of Chemistry, VŠB Technical University of Ostrava, 17. listopadu 2172/15, 700 33 Ostrava, Czech Republic

[2] Institute of Environmental technologies, VŠB Technical University of Ostrava, 17. listopadu 2172/15, 700 33 Ostrava, Czech Republic

* Correspondence: petr.praus@vsb.cz

Received: 23 July 2019; Accepted: 22 August 2019; Published: 23 August 2019

Supplementary materials

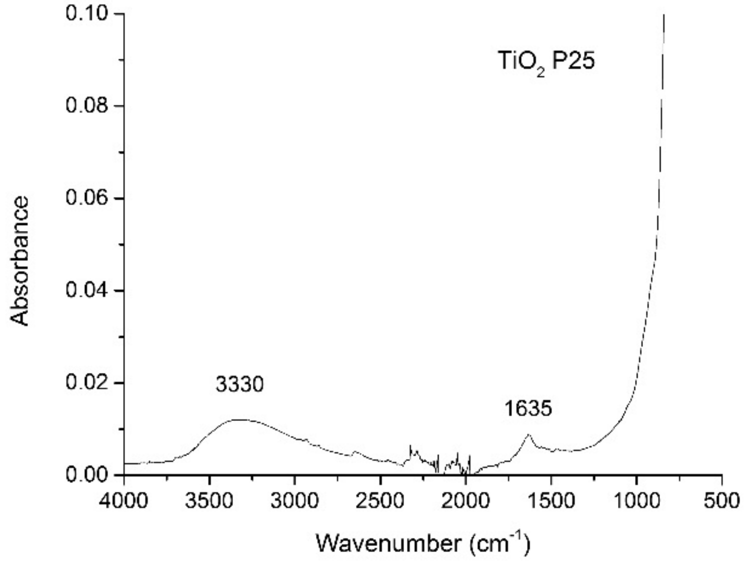

Figure S1. FTIR-ATR spectrum of TiO_2 P25.

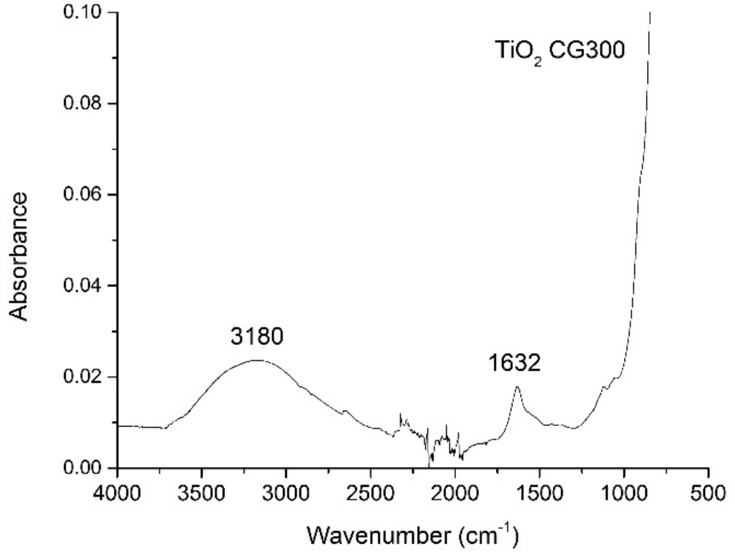

Figure S2. FTIR-ATR spectrum of TiO_2 CG300.

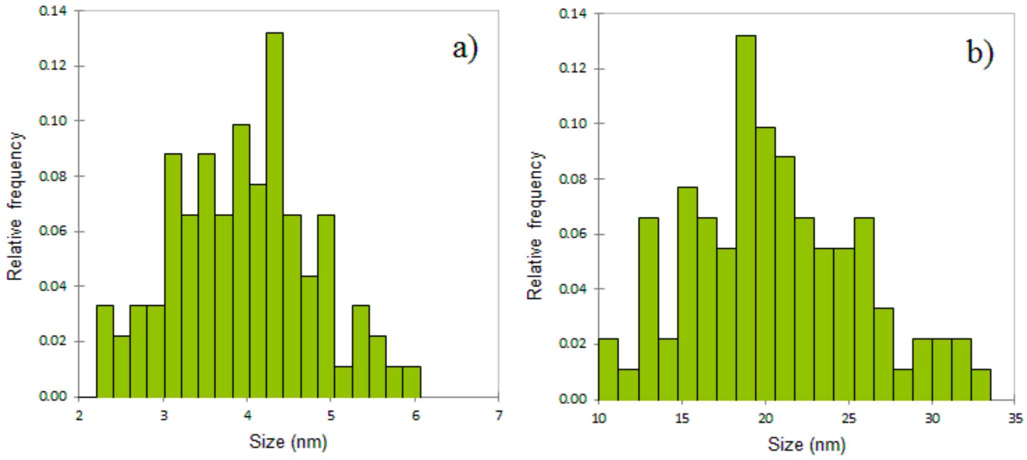

Figure S3. Histograms of sizes of TiO_2 nanoparticles. (**a**) CG300, (**b**) P25.

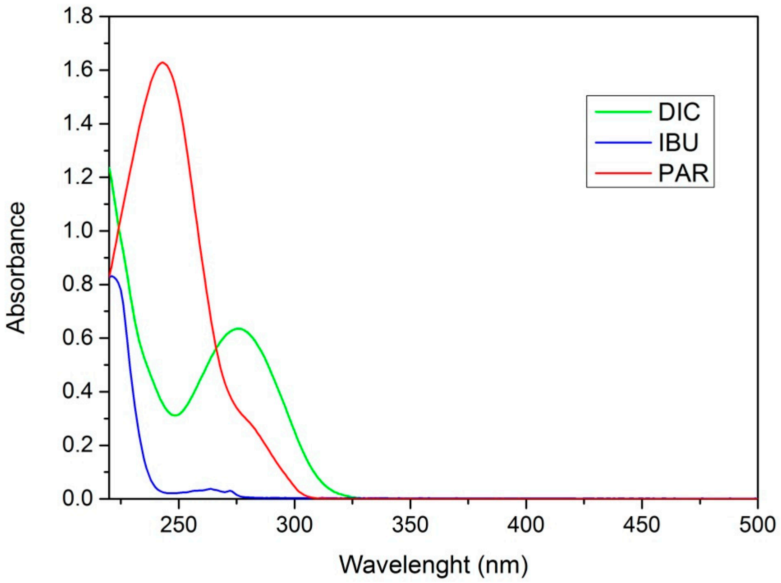

Figure S4. Absorption spectra of DIC, IBU and PAR in the concentration of 20 mg/L.

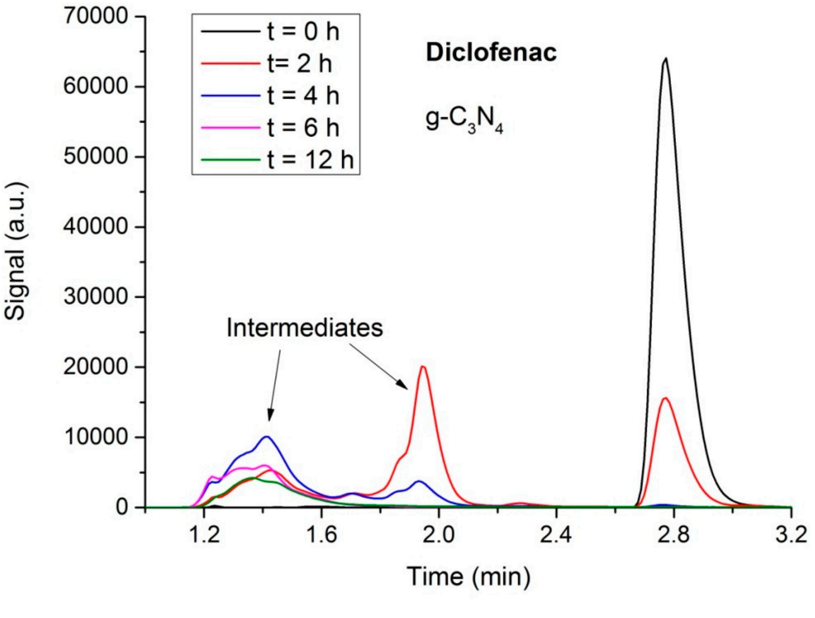

Figure S5. HPLC chromatograms of diclofenac photodegradation with g-C_3N_4
